# Household contact investigation for tuberculosis in Vietnam: study protocol for a cluster randomized controlled trial

**DOI:** 10.1186/1745-6215-14-342

**Published:** 2013-10-20

**Authors:** Gregory J Fox, Nguyen Viet Nhung, Dinh Ngoc Sy, Warwick J Britton, Guy B Marks

**Affiliations:** 1 Woolcock Institute of Medical Research, 431 Glebe Point Road, Glebe, NSW 2037, Australia; 2 National Lung Hospital, Ba Dinh, Hanoi, Vietnam; 3 Sydney Medical School, University of Sydney, Sydney, NSW 2006, Australia; 4 Centenary Institute, Newtown, NSW 2042, Australia; 5 Liverpool Hospital, Sydney, NSW 2170, Australia

**Keywords:** Contact tracing, *Mycobacterium tuberculosis*, Public health, Pulmonary tuberculosis, Screening, Tuberculosis

## Abstract

**Background:**

Tuberculosis is an infectious disease that continues to cause considerable morbidity and mortality globally. Only 65% of patients worldwide are currently diagnosed. Contact investigation is a strategy that aims to increase case detection and reduce transmission of tuberculosis, yet there is little evidence to show its effectiveness.

**Methods/Design:**

We will conduct a cluster randomized controlled trial of contact investigation within the national tuberculosis control program of Vietnam. Household contacts of patients with smear-positive pulmonary tuberculosis will be invited to attend district tuberculosis units for symptom screening, examination, and chest radiography on four occasions over a two-year period. The primary endpoint is clinically confirmed tuberculosis among contacts during the 24 months of follow-up, ascertained using capture-recapture analysis. Microbiologically proven tuberculosis and treatment completion rates among contacts diagnosed with tuberculosis will be secondary endpoints. The incremental cost-effectiveness ratio will be estimated. The study will have 80% power to detect a 50% increase in the primary endpoint in the active intervention arm compared with the control arm. The study will include 8,829 contacts in each of the active screening and control groups, within 70 districts in 8 provinces in Vietnam, in both rural and urban settings.

**Discussion:**

The effectiveness of contact investigation as a tool for improved tuberculosis case finding has not been established. This cluster randomized trial will provide valuable operational information for national tuberculosis programs in high-prevalence countries, in order to select the most cost-effective strategies to improve tuberculosis case detection.

**Trial registration:**

The ACT2 study has been registered with the Australian New Zealand Clinical Trials Registry (ACTRN12610000600044).

## Background

Tuberculosis (TB) is an airborne infectious disease that affects almost nine million people each year, mainly in resource-limited settings [1]. The predominant global strategy for identifying people with TB is 'passive case detection’, which relies upon a diagnosis of TB being made in symptomatic patients presenting to healthcare facilities for evaluation [2]. This approach is predicated upon the assumption that most patients will be symptomatic. However, despite the widespread adoption of this approach in high-prevalence countries, only 65% (63 to 68%) of the estimated total number of patients with TB are diagnosed each year [3]. Those patients who are not diagnosed are not treated, and are therefore at risk of transmitting the infection to others. If the Stop TB Partnership’s goal of global TB elimination by 2050 is to be realized [4,5], new evidence-based strategies for enhancing case detection are urgently needed.

### Rationale for the study

Recent prevalence surveys in countries with a persistently high prevalence of TB have found that a substantial proportion of disease is not detected by conventional passive case-finding strategies [6-8]. Strategies that increase the proportion of cases that are detected may interrupt ongoing transmission of *M. tuberculosis*, and reduce the prevalence of TB over time [9,10]. Case detection strategies that target high-risk populations and are integrated with national TB control programs [11] are most likely to be feasible and sustainable.

Contacts of patients with TB are known to have a high risk of developing the disease, particularly in the five years after exposure. A recent meta-analysis of studies including almost 900,000 contacts showed that 3.1% (95% confidence interval (CI), 2.2 to 4.4%) of contacts in studies from resource-limited settings and 1.4% (95% CI, 1.1 to 1.8%) of contacts in studies from high-income countries had co-prevalent TB [12]. In Vietnam, our previously reported cohort study of active case finding in tuberculosis (ACT1) found that among 545 household contacts, 0.7% had co-prevalent disease [13]. Consequently, contacts are a potentially high-yield population to screen for disease. Close contacts of TB patients are also identified relatively easily by TB control programs, which can build upon their existing therapeutic relationship with the TB patient. For these reasons, contact investigation, defined as the evaluation of contacts of known TB patients for disease or infection is potentially a worthwhile adjunct to an effective TB control program in resource-limited settings.

Although contact investigation has been standard practice in most high-income countries for many years [14-17], it has been much less commonplace in resource-limited settings. Contact investigation policies in these settings have generally focused on specific high-risk contacts, such as children [18,19] and people living with HIV [20]. Recent World Health Organization (WHO) guidelines for TB contact investigation recommended the intervention be extended to close contacts in settings where adequate resources are available [21]. However, the expert panel acknowledged that the quality of the available evidence for the practice was very poor, according to GRADE criteria [21]. Although many published studies report a high yield among contacts, few assess the effectiveness compared with standard passive case finding [22]. There have been few randomized controlled trials of household contact investigation published to date [23,24].

Strategies for enhanced case detection in high-prevalence settings must be feasible and cost-effective, so that they are appropriate and affordable in the local context. It is important that countries considering implementing contact investigation have a high rate of DOTS coverage and consistently high treatment completion rates, so that additional cases identified by enhanced case detection are likely to be treated successfully [25,26]. In order to be informative to health policy makers, this study is designed so that the intervention that is being evaluated can be implemented and scaled up within the busy and resource-limited environment of the Vietnamese healthcare system.

### The research context

Vietnam is a middle-income country that is ranked eleventh in TB burden among the high-prevalence countries. A national survey in 2006 estimated that the prevalence of TB was 307 bacteriologically proven cases per 100,000 adults (95% CI, 249 to 366 per 100,000 persons aged 15 and over) [7]. Despite consistently achieving WHO targets for treatment completion rates since 1998, annual case notifications have not yet declined. Based on data from the prevalence survey and routine reporting, the WHO estimates that only 56% (95% CI, 44 to 73%) of all cases are diagnosed [1]. These data suggest that the Vietnamese National Tuberculosis Program (NTP), which oversees the majority of TB control in the country, may need to consider new approaches to identify disease in the population.

The Active Case Finding of Tuberculosis (ACT2) study is a cluster randomized controlled trial of contact investigation that is conducted by staff of the NTP, based at local district health facilities, and integrated within the management structure of the NTP.

### Hypothesis and aims

The hypothesis of this study is that, in countries that are highly endemic for TB, targeted active case finding among household contacts will result in more contacts being diagnosed with TB than will standard passive case finding alone.

The primary aim of the study is to determine if household contact investigation is effective, by determining whether it increases the overall yield of active TB diagnosed among household contacts of patients with smear-positive TB compared with usual passive case detection. There are two secondary aims. The first is to estimate the cost effectiveness of this intervention by measuring the incremental cost-effectiveness ratio. The second is to determine the feasibility of implementing contact investigation within the NTP in a range of settings in Vietnam, including urban and rural environments. The screening strategy proposed in this study will be operationally nested within the NTP.

## Methods/Design

This is a cluster randomized controlled trial with two arms comparing standard passive case detection with household contact investigation in eight provinces in Vietnam. In the active intervention clusters, household contacts of patients with TB will be assessed for the disease four times over a two-year period. The assessment comprises clinical evaluation, chest radiography and, in TB suspects, microbiological testing of sputum.

### Inclusion and exclusion criteria

All patients aged 15 years or over with pulmonary TB and at least one sputum sample positive for acid-fast bacilli on direct smear microscopy will be eligible to enter the study. Patients are excluded if they have no eligible household contacts.

Household contacts of any age are eligible for inclusion if they lived in the same dwelling as the index patient during the two months prior to the diagnosis of the index patient. Pregnant women are eligible for inclusion, but women known to be pregnant are not offered a chest radiograph.

### Study endpoints

The following endpoints are being measured in all contacts (in both groups). The primary endpoint is the proportion of contacts with clinically diagnosed TB. This will be ascertained based on the results of screening (in the active screening districts) and routine notifications to the NTP (in the active screening and control districts). Secondary endpoints, as indirect measures of study feasibility, are: (a) the weighted proportion of contacts with microbiologically proven TB (defined as either smear or culture positive TB), smear-positive TB and extrapulmonary TB; (b) cost effectiveness, determined by calculating the direct and indirect costs for each additional case diagnosed; and (c) the proportion of eligible contacts enrolling in the study, and the proportion of contacts participating in scheduled follow-up.

### Description of intervention

#### *Recruitment of index cases*

Index cases with smear-positive pulmonary TB and their contacts are identified in both active and control study districts. The study intervention is only implemented for contacts in the active intervention districts.

Consecutive patients with smear-positive pulmonary TB, who live in the study districts, are identified by TB staff working within district hospitals or district tuberculosis units. Those who agree to participate are given a participant information statement and asked to sign a written consent form.

#### *Recruitment of contacts*

Contacts are identified using a screening questionnaire completed in consultation with the index case. Household contacts include all members of the household occupied by the index case, including children and infants, who were present during the period of recent infectivity, that is, two months up to the time of diagnosis. Household contacts are approached by the index patient in their family and given information explaining the study. Contacts are invited by the patient to enroll in the study at a district clinic.

#### *Active intervention*

All contacts in the active intervention districts will be evaluated at the time they are first identified, and then 6, 12, and 24 months post-exposure. The procedures will be free of charge to the participants. On each occasion, the assessment will include a symptom questionnaire, physical examination, and chest X-ray. Contacts will have a chest X-ray at a local clinic or nearby private radiology service. X-ray films will be read by local clinical staff for routine clinical action and then be transferred to a central location for standardized re-reading by provincial level staff.

Contacts will be defined as having possible TB if they have: (a) cough lasting for two weeks, (b) sputum for two weeks, (c) any hemoptysis, or (d) an abnormal chest X-ray meeting the criteria for possible TB. Contacts meeting one or more of these criteria will be asked to submit two spontaneous sputum specimens collected on consecutive days for acid-fast bacilli (AFB) examination. Two direct smear examinations and one culture test will be undertaken in the Provincial Hospital laboratory. For children younger than five years, the diagnosis of TB will be made on clinical and radiological grounds, in accordance with local policy at the time of commencement of the study. Treatment of active disease will be according to the policies of the NTP. An experienced clinician in each province will be available to advise district staff regarding the diagnosis of childhood TB and the investigation of suspected extrapulmonary disease.

### Control districts

Contacts enrolled in control districts, will be given general information about the typical symptoms of TB and advised to report to the district hospital or TB unit if they develop these symptoms. During the study period, self-referring contacts with TB, who are participating in the study, will have two sputum specimens transferred to the reference laboratory for culture, in addition to any routine diagnostic procedures. This will allow comparison of rates of culture-confirmed TB between active and control districts. Contacts diagnosed with TB in both the control and active intervention groups will receive standard treatment for TB through the NTP.

### Selection of study districts

The study will be conducted in 70 districts within eight provinces. These provinces have been selected to include four provinces in the south (Ho Chi Minh City, Can Tho City, and the rural provinces of An Giang and Tien Giang), where the prevalence of TB is highest, two provinces from the central area (Da Nang Province and the rural province of Binh Dinh), and two provinces from the north (Hanoi City and the semi-rural province of Vinh Phuc). This geographic and urban-rural stratification is important because the incidence of TB increases from the north to the south within Vietnam [7]. There are also substantial differences in lifestyle and infrastructure between rural and urban areas. Hence, the selection of this diverse range of sites will increase the generalizability of the study findings.

### Randomization

Among the 97 districts within these eight provinces, 70 have been randomly selected for participation in the trial. These districts were selected with a probability proportional to their population size. The initially selected districts will enter a run-in period, prior to randomization, during which time they will be required to recruit two patients and six contacts and to demonstrate that they have the necessary resources to conduct the study. If districts fail or withdraw during this pre-randomization run-in period, they will be replaced by the next randomly selected district.

We will randomly allocate districts to the active intervention or control group using the minimization method with predefined randomization lists [27]. The minimization criterion will be province, to ensure balance of intervention and control districts within each province. District size will not be taken into account in the randomization process. Randomization will be performed by a person who is not otherwise engaged in the project, and is blinded to the identity of districts.

### Sample size calculation

Based on an earlier meta-analysis, we estimated that the prevalence of confirmed active TB in close contacts of smear-positive cases would be 2.3% [28]. In the 2006 to 2007 National Prevalence Survey, approximately 50% of prevalent cases of TB did not report typical symptoms of the disease, suggesting that a substantial proportion of patients with prevalent disease are not diagnosed by routine passive case detection within the NTP. We estimate that screening with the combination of X-ray and symptoms will identify at least 75% of prevalent cases of active pulmonary TB. This represents an increase in case detection rate of 50% above that expected for passive case finding alone. To have 80% power to detect an effect of this size, we would need 3,313 contacts in each group (6,626 in total), without accounting for clustering [29].

The sample size was then adjusted for the clustered design of the study. The first level of clustering is at the level of the index case. We estimated that, on average, each index case would identify four close contacts who would consent to participate. Assuming that the intraclass correlation coefficient within households is 0.1, the design effect at this level will be 1.3. The second level of clustering is at the level of the districts. There were 152 cases of smear-positive pulmonary TB per district in 11 representative provinces in 2007. Assuming that 70% of smear-positive cases consent to participate in the study, there will be 106 index cases. If we assume an intraclass correlation coefficient for index cases within districts of 0.01, the design effect at this level will be 2.05. Hence, we anticipate an overall design effect of 2.67.

On this basis, we estimate that we need to recruit a total of 3,338 index cases and 8,829 contacts (of 2,207 index cases) in each group. Based on our assumptions, this would require us to approach all available patients diagnosed with smear-positive pulmonary TB in 21 districts in each group: 42 districts in total. To ensure that we have a conservative sample size calculation, we will recruit all eligible and consenting index cases and their contacts from 70 districts.

Specific quarterly recruitment targets will be set for each district, based upon the number of notified cases of smear-positive TB during the most recent year for which data were available, as a way of enabling districts to track their progress during the study.

### Analysis of effectiveness

The effectiveness of the active case finding in contacts will be assessed by comparing the case detection rate between contacts of index cases in the active intervention and control districts. There are two levels of clustering in the selection of contacts: the district in which the contact lives and the index case with whom he or she was in contact. To account for these two levels of clustering, we will undertake a mixed model analysis in which districts and index cases within districts are random effects and active versus control status is the fixed effect. The measure of effect will be relative risk, estimated using a log link and binomial error distribution. In this primary analysis, there will be no adjustment for baseline characteristics. However, in a subsidiary analysis we will examine the impact of baseline characteristics of the districts, including geographical characteristics and baseline case notification rate, and individuals, including age and sex, on the risk of disease by testing these characteristics as potential effect modifiers.

### Primary and secondary endpoints and stratification

The primary endpoint will be the weighted proportion of contacts with clinically diagnosed TB in screening and control districts during the two-year follow-up period of the study. Secondary endpoints will include the weighted proportion of contacts with microbiologically proven TB, smear-positive TB, and extrapulmonary TB. We will also estimate the proportion of contacts with TB who complete treatment. Stratum specific weighted prevalence rates of clinically diagnosed and microbiologically proven TB will be determined for region and rurality.

### Analysis of cost effectiveness

Direct costs of diagnosis and treatment will be determined based upon questionnaires for staff and patients at provincial and district level healthcare facilities. Indirect costs will be determined based upon detailed questionnaires for patients, contacts, and staff. Costs will be classified into one of two categories: those related to the identification of new TB suspects among the contacts of TB cases and those related to investigation and management of TB suspects. The former are costs that are unique to this intervention. The latter costs are already met by the NTP but these will be increased by the intervention because there will be more TB suspects.

The incremental cost-effectiveness ratio will be calculated as the difference in total cost for TB control measures between the active and control districts divided by the difference in the number of cases detected.

### Data collection and management

Data will be recorded on paper forms at district clinics and provincial hospitals. Each district will submit a monthly report on the recruitment index cases and contacts and the occurrence of study endpoints. These data will be entered within one month of collection into an online database. Written data and chest radiographs from each district will be digitally photographed, and the images transferred to the National Lung Hospital for electronic data entry and quality control. Data will be entered using a custom-designed, internet-based database, and monthly data checking and quality control will be performed at a central level. District staff will be provided regularly with a list of additional information required. A record of the proportion of children diagnosed with TB by chest X-ray will be compiled every three months, to ensure that the proportion of children receiving tuberculosis treatment does not exceed that recognized in the published literature.

### Quality control

The technical quality of chest radiographs will be assessed at the National Lung Hospital by examining all radiographs labeled as abnormal and a random sample of all other radiographs. All radiographs read at the district level will be re-read at the provincial level, and the district staff will be informed of discrepancies. Quality control of laboratory testing will be performed according to the routine protocols of the NTP, including structured quarterly monitoring visits by staff from the National Lung Hospital.

### Evaluation and monitoring

Members of the project research staff will conduct monitoring visits to each province every quarter, and to each district at least once each year. Provincial TB control staff will also conduct quarterly monitoring visits to each district in conjunction with routine monitoring for the NTP. Monitoring visits will include checking participant consent forms, confirmation that enrolled index patients have been included in the district notification registry, checking research forms for completeness, and compliance with study protocols.

To verify subject consent, randomly selected contacts from each district will be telephoned by study staff to confirm they have consented to participate in the research. Irregularities in recruitment will be followed up by provincial staff and reported back to research staff.

### Ethical issues

#### *Ethical approval*

The protocol, patient information sheet, and patient consent form have been approved by the Human Research Ethics Committee at the University of Sydney, the Scientific Committee of the Vietnam Ministry of Health and the Scientific Committee of the Vietnam National Lung Hospital.

#### *Informed consent*

Subjects may only enter the study if written informed consent is obtained. Written consent by parents will be required for children under 15 years of age. The healthcare worker enrolling the subject will be responsible for obtaining written informed consent and consent forms will be checked during regular monitoring visits by research staff.

#### *Withdrawal from the study*

Research participants may voluntarily withdraw from the study for any reason. If they withdraw then there is to be no adverse impact upon the treatment of the TB patient within the NTP.

#### *Confidentiality*

A unique study code will be assigned to each research subject, and used to identify radiographs and laboratory specimens. Written records will be stored securely in the district clinics and provincial hospitals or at the National Lung Hospital. The online database and electronic records will be password protected.

## Discussion

Investigation of TB contacts may be a valuable next step in controlling TB in countries that have achieved consistently good treatment outcomes under directly observed therapy (DOT). Household contacts of TB patients are recognized to have a high risk of disease [12]. However, there is not yet sufficient evidence to determine whether active case finding in contacts will detect more cases than passive case finding alone [22]. Furthermore, there are no data on the cost effectiveness of contact investigation in Vietnam. Consequently, a randomized controlled trial is required to assess the effectiveness and cost effectiveness of contact investigation in a high-prevalence setting.

This study will be limited to contacts of smear-positive patients because smear-positive index patients are more likely to transmit TB [30] and the prevalence of TB among their contacts is high [12]. Since prolonged exposure to an infectious patient confers a higher risk of disease [31,32], household contacts form an ideal population to target for screening.

There is limited evidence quantifying the extent to which disease among household contacts is actually caused by transmission within the household. Several studies in high-prevalence settings have shown that between 17% and 46% of households where two or more patients have TB share identical molecular fingerprints [33-35]. This suggests that much transmission may occur outside of the household. However, these studies may have underestimated household transmission, as they only included patients diagnosed through passive case finding. As a result, they may have missed household contacts with minimal symptoms. Nonetheless, household contacts will also share other environmental and genetic risk factors for disease with the index patient. Contacts are also relatively accessible to TB treatment programs that are already treating the index patient.

Importantly, this research is closely integrated with the existing national TB control program. This integration will ensure that experienced workers within the NTP assess contacts, and that affected contacts can readily be treated. This research design is essential to the general applicability of the research and its relevance to future policy decision-making and scale-up. The study is a form of operational research, which is a systematic approach to developing evidence for an intervention that is useful to policy makers [36]. Operational research is a cornerstone of the WHO’s Stop TB strategy, since high quality trial evidence is recognized to be essential if limited health resources are to be directed most appropriately [37]. By integrating research within the Vietnamese NTP, the outcomes of this study will be relevant and generalizable beyond the research setting. A limitation of our approach is that we will not be administering isoniazid preventive therapy (IPT) to child contacts. This approach is recommended by international guidelines [38]. At the time that recruitment to this study commenced, IPT was not the standard of care for child contacts in Vietnam. Therefore, based on the local policies at the time of study commencement, clinical and radiological evaluation will be the cornerstone of our evaluation of children. National Guidelines were revised in 2012 to recommend IPT for child contacts of patients with TB, with implementation over the following year. Recruitment to this study was due to end by mid-2013, hence it was not possible to change practice for IPT for contacts in the context of this study. Future contact tracing investigations in adults in Vietnam should include prescription of IPT for child contacts. By performing serial chest radiographs for contacts in the screening group until 24 months, we will be able to detect and treat a high proportion of disease that develops in this time.

Although randomized controlled trials have often been considered overly ambitious in resource-limited settings, our study will combine the rigor of this design with the practicalities of routine TB control. It will also contribute to the capacity of local staff to conduct research, and support the supervision of the research within the structures of the NTP. By implementing the study within eight provinces, we will also learn valuable lessons about the practical challenges encountered in contact investigation throughout the country that will inform future policy development.

Therefore, this cluster randomized controlled trial will provide an important evidence base to assist policy makers in Vietnam and comparable countries in their decisions about the programmatic implementation of active case finding in the future.

## Trial status

Currently recruiting.

## Abbreviations

AFB: Acid-fast bacilli; CI: Confidence interval; DOTS: Directly observed therapy short-course; IPT: Isoniazid preventive therapy; NHMRC: National health and medical research council; NTP: National tuberculosis program; TB: Tuberculosis; WHO: World Health Organization.

## Competing interests

The authors declare that they have no competing interests.

## Authors’ contributions

GBM and GJF developed the study methods, drafted the manuscript, revised it for important intellectual content, and approved the final version. They currently provide management and oversight of the trial. GJF supervised staff implementing the research, and monitored and evaluated the research. GBM and WJB wrote the grant application to obtain funding. NVN and DNS provided intellectual input into the development of the study methods, supervised staff implementing the research, and obtained ethical approval for the research. They approved the final version. WJB contributed to the development of study methods, drafting the manuscript and revising it for important intellectual content, and provided approval for the final version. All authors reviewed and approved the final manuscript.

## Authors’ information

GJF is a Respiratory Physician and Research Fellow at the Woolcock Institute of Medical Research. He was Country Director for the Woolcock Institute of Medical Research during the establishment of the trial. NVN is the Vice-Director of the National Lung Hospital and the NTP. He is an Associate Professor of Respiratory Medicine at Hanoi Medical University. DNS is the Director of the National Lung Hospital and the NTP. He is an Associate Professor of Respiratory Medicine at Hanoi Medical University. WJB is the Bosch Professor of Medicine and Professor of Immunology at Sydney Medical School, University of Sydney. He is the Director of the NHMRC Centre for Research Excellence in Tuberculosis in Control, Australia, and is also the head of the Tuberculosis Research Program at the Centenary Institute for Cancer Medicine and Cell Biology at the University of Sydney. GBM is a Clinical Professor at the University of Sydney and the Head of Respiratory and Environmental Epidemiology at the Woolcock Institute of Medical Research, Sydney. He is also a Senior Staff Specialist Respiratory Physician at Liverpool Health Service, Sydney.
